# Breast Cancer Genetic Counseling: A Surgeon’s Perspective

**DOI:** 10.3389/fsurg.2016.00004

**Published:** 2016-01-28

**Authors:** Doreen M. Agnese, Raphael E. Pollock

**Affiliations:** ^1^Division of Surgical Oncology, Department of Surgery, The Ohio State University, Columbus, OH, USA

**Keywords:** breast cancer, genetic risk assessment, genetic counseling, genetic testing, hereditary breast cancer

## Abstract

As surgeons who care for patients with breast cancer, the possibility of a cancer diagnosis being related to a hereditary predisposition is always a consideration. Not only are we as surgeons always trying to identify these patients and families but also we are often asked about a potential hereditary component by the patients and their family members. It is therefore critical that we accurately assess patients to determine who may benefit from genetic testing. Importantly, the potential benefit for identifying a hereditary breast cancer extends beyond the patient to other family members and the risk may not be only for the development of breast cancers, but for other cancers as well. This review was written from the perspective of a surgeon with additional training in cancer genetics in an effort to provide a unique perspective on the issue and feel that a review of some of the more practical considerations is important.

## Introduction

As surgeons who care for patients with breast cancer, the possibility of a cancer diagnosis being related to a hereditary predisposition is always a consideration. Not only are we as surgeons always trying to identify these patients and families but also we are often asked about a potential hereditary component by the patients and their family members. It is therefore critical that we accurately assess patients to determine who may benefit from genetic testing. Importantly, the potential benefit for identifying a hereditary breast cancer extends beyond the patient to other family members and the risk may not be only for the development of breast cancers, but for other cancers as well. This review was written from the perspective of a surgeon with additional training in cancer genetics in an effort to provide a unique perspective on the issue and feel that a review of some of the more practical considerations is important.

**Figure 1:**
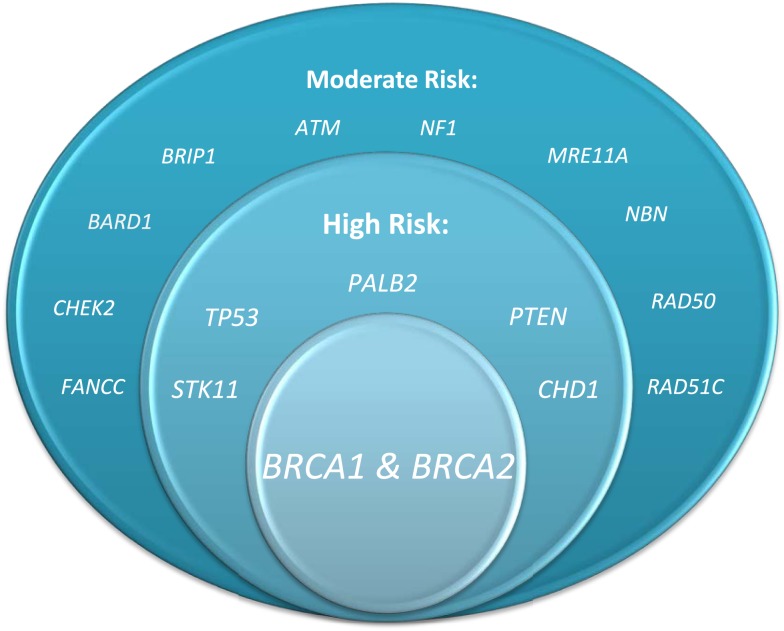
**Genes associated with elevated breast cancer risk (figure courtesy of Alexandra Suttman, BA, genetic counseling student, The Ohio State University)**.

Hereditary cases of breast cancer have long been recognized to occur, but the ability to test for a marker of risk is a relatively recent occurrence. The first study demonstrating evidence of an autosomal dominant pattern of inheritance for breast cancer was published in 1988 (1). Soon after, linkage to a gene on chromosome 17 was established, and the BRCA1 gene was identified (2, 3). Shortly after that, a second breast cancer susceptibility gene, BRCA2, was identified (4). By 1996, the first molecular test for hereditary breast and ovarian cancer was introduced by Myriad Genetics. This test was initially performed on a blood sample and consisted of sequencing the BRCA1 and 2 genes. Over the years, the test has improved to detect deletions and rearrangements that may have been missed with standard sequencing and can now be performed on either a blood or buccal sample. Additional genes have also been associated with increased risk for breast cancer (Figure 1), and new technology, referred to as next generation sequencing, has allowed for testing for mutations in numerous genes with a single blood sample.

## Risk Assessment

There are many instances where a patient’s risk can be assessed. Certainly, when assessing a newly diagnosed breast cancer, the age of onset of the cancer, specific biomarkers, and family history assessment may indicate concern for hereditary risk. Identifying unaffected individuals with hereditary risk is also of critical importance, whether they are identified due to a known genetic mutation in a family member or due to suggestive family history. The US Preventive Services Task Force has published recommendations regarding risk assessment, genetic counseling, and genetic testing for BRCA-related cancers in women (5).

It is well established that when evaluating a new patient, a family history is obtained, but often many of the important features necessary to identify a family at hereditary risk are not assessed. When assessing a family for hereditary risk, a complete history should be obtained to include three generations and note all cancer diagnoses and ages of diagnosis. The comprehensiveness of family cancer history assessments and cancer risk assessment in primary care has been investigated by Murff and colleagues (6, 7). In the first report, a retrospective chart review, the authors reviewed the charts of 995 new patent visits to 28 primary care providers to evaluate the completeness of family history of colon or breast cancer. Of the entire sample, 7% contained no documentation of any family history information. Cancer family history information was only collected on 68% of the patients, and specific information regarding the affected individual and diagnosis was only present in 61% of the records. Age at diagnosis in first degree relatives was documented in 38% of breast cancers and 27% of ovarian cancers. Only 17% of those who met criteria for early onset breast cancer were identified and referred for genetic testing services (6). In a later study, these investigators compared family history information collected through self-completed surveys to the documentation in the chart. Once again, age was often missing on chart review (recorded in 40%) but was much more commonly reported in the survey (81%) (7). The utilization of these surveys, the authors concluded, may address some of the limitations to obtaining an appropriate history in a primary care office, including limited provider time, competing clinical diagnoses, provider–patient communication issues, and lack of both provider and patient education regarding the importance of family cancer history on cancer risk assessment.

In an elegant study by Burke and colleagues, primary care physicians were presented three different unannounced standardized patient case scenarios (moderate risk, high-risk maternal and high-risk paternal, and all audiotaped), and at the conclusion, were asked to complete a brief detection questionnaire. Similar deficits in completeness of family history were identified, and again age of onset was often not assessed. Physicians did least well identifying risk due to paternal family history and some physicians’ comments ascribed less significance to the paternal family history of breast cancer than the maternal family history. Many did elicit or recognize the important contribution of ovarian cancer to hereditary breast cancer risk assessment (8). This study demonstrates a lack of understanding of the autosomal dominant inheritance pattern of hereditary breast and ovarian cancer syndrome and the importance of paternal family history and ovarian cancer history.

Even in specialized clinics, in more contemporary settings, identification of high-risk patients has been difficult. Ow and colleagues retrospectively reviewed over 6000 pedigrees at their center in Singapore over a 10-year period to identify those with over a 10% risk of having a BRCA mutation. Of the 615 identified, 506 had medical records available for review, and they found that a good family history spanning three generations was documented in only 54% and was taken less frequently than smoking and drinking history. On univariate analysis, they found that taking a good family history and young age of onset of cancer increased the likelihood of physician suspicion (9).

Patient issues also play a role in failure to identify patients at risk for hereditary cancer. Even when the appropriate questions are asked, the quality of family history data is dependent on the accuracy of patient reporting. In a study of over 43,000 women presenting for screening mammogram, Ozzane and colleagues reviewed the reported numbers of breast, colon, prostate, lung, and ovarian cancers and compared those reported in the maternal and paternal relatives and in first- and second-generation relatives. Self-reported family histories were significantly influenced by bloodline and degree of relative affected, with lower reporting of all cancers with the exception of prostate cancer in the paternal lineage and in second degree relatives (10).

## Counseling and Testing Issues

Once an accurate personal and family history has been obtained, the issue of genetic testing can be explored, but when should the test be ordered, who should order the test and how should results be communicated? Individuals at risk for hereditary cancer can be identified at multiple different times. They may be identified after diagnosis and definitive treatment of breast cancer, after diagnosis but before definitive treatment, and when unaffected. Each scenario presents its own set of challenges and different approaches to the patients with respect to counseling, testing, and risk management are required (11). Since the testing is expensive, insurance coverage can be a concern. Most insurance companies have established criteria for coverage of genetic testing based on the National Comprehensive Cancer Network’s guidelines (12). Some of the newer panel tests are not covered by some insurers.

For unaffected individuals at risk, positive results will help to quantify risk. Generally, options for management of risk include surveillance with regular mammography and clinical examination and in some cases, MRI, chemoprevention, and risk-reducing surgery. Negative genetic test results, however, may not completely eliminate risk. Without testing an affected individual, it is not clear if a negative result indicates population risk, because the mutation in the family was not inherited, or persistent risk, because the genes tested for are not responsible for the cancers in the family. For those with a new diagnosis who have not yet had definitive surgery, genetic testing results may influence surgical decisions. For example, if a patient tests positive for a mutation in one of the BRCA genes, she may choose to have bilateral mastectomy due to increased risk of contralateral cancer compared to non-mutation carriers. Identification of other mutations less well described than BRCA1 and 2 may not influence surgical decisions, since less information regarding contralateral cancer risks is known. The timing necessary for counseling and testing in the perioperative period may lead to delays in care or may cause additional anxiety if results are not obtained prior to surgery. In our institution, surgical decision-making cases are generally seen within 1 week, but extent of testing will impact timing of results availability. If only BRCA testing is performed, results are generally available within 1 week, but if a panel of genes is tested, results can take 4–6 weeks.

Regardless of the timing of testing, the growing complexity of genetic testing options, including testing for panels of genes for which less information is available, make pre-test and post-test counseling important components of the process (12). Pre-test counseling should include an assessment of risk and a discussion of the differential diagnosis along with education of the inheritance patterns, penetrance, and variable expressivity. Pre-test counseling in general is associated with improvement in cancer-specific knowledge and minimal adverse psychological consequences (13). Direct to consumer testing has emerged as another option for patients. This refers to genetic testing marketed directly to consumers via a variety of methods, including television, print ads, and the internet and does not necessarily involve a physician or insurance company. There is little to no counseling performed prior to testing. The results are shared directly with the consumer. In some cases, a genetics professional may be involved in explanation of the results. There are significant risks and limitations to these tests, as consumers may be misled by unproven tests and without guidance from a physician or counselor, they may make important health care decisions based on inaccurate or incomplete information. In spite of these concerns and limitations, available published data suggest that clear benefit was provided to participants without serious emotional distress or inappropriate actions (14). Women with breast cancer even preferred testing without prior counseling and did not experience increased distress (15). It is important to note, however, that the current body of literature regarding direct to consumer testing predated panel testing, which certainly does complicate the interpretation of results.

When pre-test counseling is performed, it is generally performed by a genetic counselor, medical geneticist, surgical or medical oncologist, or other health professional with expertise and experience in cancer genetics. Some insurance companies, including Cigna and United Health Care, require that counseling be provided by a certified genetic counselor prior to testing. The American Society of Clinical Oncology recently published a policy statement update on genetic testing for cancer susceptibility. The recognition and management of individuals with an inherited susceptibility to cancer is considered a core element of oncology care, and as such, continued education of oncologists and other health professionals in the area of cancer risk assessment and management of individuals with an inherited predisposition to cancer is recommended (16). The key factor is expertise and experience in cancer genetics. Educational courses and self-teaching tools are available through City of Hope and ASCO.

Cragun and colleagues assessed the potential differences in genetic counseling and testing services between board-certified genetic health-care providers and non-genetic health care providers by surveying patients who had undergone testing. They evaluated patient recall and content of precounseling for hereditary breast and ovarian cancer and whether BRCA1 and 2 gene sequencing was performed when less expensive single site or Ashkenazi Jewish founder mutation testing may have been sufficient. They found that genetic health-care provider involvement was associated with not only adherence to nationally recommended genetic counseling practices but could also reduce cost of testing (17). Vadaparampil and colleagues examined whether non-genetics professionals in Florida performed guideline-based patient intake and informed consent before genetic testing. They surveyed 386 non-genetics providers offering BRCA testing in the state of Florida. Of the 81 respondents to the survey, few constructed three-generation pedigrees, discussed alternative hereditary cancer syndromes or discussed the meaning of a variant result. Approximately half reported sometimes scheduling a separate session for pre-test counseling, discussing family implications of testing, benefits and limitations of risk management options, and discussion of the potential psychological impact and insurance-related issues. This lack of adherence to established guidelines may result in harm to patients and their families, including ordering the wrong test, negative emotional effects, receiving incorrect medical management guidelines, and misinterpretation of results, leading to wasted health care resources and unnecessary preventive surgeries. In instances where the non-genetics provider cannot provide these services, referral to a genetics professional is encouraged (18), but as noted above, non-geneticists who learn the tools can appropriately counsel patients.

Testing of affected individuals who have already been treated for their cancer is routinely performed and is historically the way testing was initiated in families with hereditary risk. With the growing public knowledge regarding hereditary forms of breast cancer, newly diagnosed patients who have not yet had treatment often wonder about their hereditary risk and contemplate testing. Testing could be considered once the primary tumor has been treated, but is often being considered prior to definitive therapy. Concern exists regarding the appropriateness of genetic counseling and testing in newly diagnosed patients who have not yet had definitive surgery. This testing may have important implications for surgical decision making, including consideration of contralateral prophylactic mastectomy or the use of certain adjuvant systemic therapies, such as PARP inhibitors and platinum compounds (19, 20), but the priority of care should be appropriate treatment of the malignancy already diagnosed rather than one that may or may not occur in the future. In a study evaluating those with breast cancer who had pre-test counseling, cancer-related knowledge was increased for all. For those counseled before definitive surgery, distress was lowered, but increased decisional conflict related to the decision for genetic testing was noted, although this was not statistically significant; for those counseled after definitive surgery, informed decision making was improved (21). In another study, newly diagnosed breast cancer patients were randomized to a group offered rapid genetic counseling and testing and a usual care control group. Two hundred sixty-five patients were recruited over a 2-year period, 178 of whom were in the intervention group. Genetic counseling was performed in 177, of whom 71 (40%) had rapid genetic testing and 59 (33%) received results prior to surgery. In this study, those who had rapid genetic counseling and testing did not have any measurable adverse psychosocial effects, including cancer worries, cancer-specific distress, anxiety and depression, cancer-specific health-related quality of life, and satisfaction with decision making and decisional conflict (22).

For unaffected women with a strong family history of breast cancer, genetic counseling is also valuable. A Cochrane review of genetic risk assessment for individuals at risk of familial breast cancer was published in 2012, and although this review found favorable outcomes for patients after risk assessment, there were too few papers to make any significant conclusions about how best to deliver services (23). Fear of developing breast cancer is often experienced by women with a strong family history of breast cancer, and many of these women may consider preventive surgery based on their perceived risks. Genetic counseling may help to more accurately quantify risk to allow women to make a more informed choice regarding screening and prevention, even if testing is not ultimately performed. In one study, a total of 83 women were referred for genetics evaluation based on perceived breast cancer risk. Of these, 31 were considering risk-reducing surgery, 23 of whom were thought to have an elevated risk of having a BRCA mutation. Ten women were tested, and a mutation was discovered in five. After counseling and testing, only 18 (58%) proceeded with risk-reducing surgery, highlighting the lack of clear understanding of risk in the medical and lay community (24). Both carriers and non-carriers of mutations derive benefit from the testing process, although women with a positive result may have a sustained increased in breast cancer distress following results disclosure without other adverse psychological outcomes (25). Fear exists, particularly among unaffected individuals, that genetic test results will be somehow used against them. Despite the passage of the Genetic Information Non-discrimination act (GINA), patient and provider awareness of legal protections for genetic testing remains low, leading to continued fears of genetic discrimination (26). GINA prohibits employers and health insurance companies from discriminating against an individual based on his or her genetic information. Specifically, genetic test results cannot be used to affect premium rates, deny coverage or make employment determinations. GINA does not, however, protect against life insurance discrimination.

Once counseling is completed, the genetic testing options should be considered, and risks, benefits, and limitations of the testing options discussed. Once this is complete, testing can be ordered and a plan should be made for disclosure of results. The options for testing have expanded in the last 2 years for two major reasons. The patent held by Myriad Genetics was overturned, enabling other laboratories to offer BRCA testing and newer technology (next generation sequencing) became available, allowing more extensive testing from a single blood sample. Testing options currently include testing only for BRCA1 and 2 mutations, testing for a panel of high-risk breast cancer genes, including BRCA1 and 2, and more extensive panels including moderate risk genes and limited evidence genes. Testing with a panel seems most appropriate when there is concern for more than one hereditary condition. For example, in a family with early onset breast cancer (under the age of 35), ovarian cancer, and early onset colon cancer, panel testing would be ideal, since potential responsible genetic syndromes include Hereditary Breast and Ovarian Cancer Syndrome, Li Fraumeni syndrome, and Lynch Syndrome.

Results interpretation is not always straightforward. For the high-risk genes, cancer risks and management options are fairly well established. The moderate risk genes have generally been seen more frequently in individuals with a strong family history of breast cancer in whom BRCA testing was negative, but the exact cancer risks and management options are not clear. It is also not clear whether these gene mutations are sufficient to explain the family history themselves or if, more likely, other modifier genes impact the likelihood of cancer development. It is therefore still debated among genetic professionals whether or not predictive testing of unaffected individuals is appropriate. In addition, a number of different testing results are possible. It is possible that a deleterious mutation in a well-described gene could be identified. This would mean that the affected individual is at increased risk for the development of cancer and that family members are at risk. The result could be negative for a deleterious mutation, but the implications of this negative result will vary based on the individual tested and the family history. An unaffected individual who tests negative for a known mutation in a family can be reassured by these negative results, as their cancer risks will be more closely aligned with population risk. Results can also be uninformative. An affected family member who tests negative for only BRCA1 or BRCA2 may have another hereditary condition not tested for. In an unaffected person with strong family history and no prior testing in the family, risk may still be elevated based on family history, but could be reduced to population if another family member was found to carry a mutation. An even more potentially confusing result is a variant of uncertain significance, or VUS. These variants are usually relatively minor changes in the genetic code for which insufficient evidence exists to determine their relevance. Decisions about risk management should not be based on these results, but rather on the family history. These unclear results may be eventually reclassified by tracking their presence with those affected with cancer in the family or with further functional studies, but this process can take years. In addition, since the contribution of these changes to cancer risk are not clear, predictive testing is not recommended for other at risk individuals. This is an area of potential harm to patients, since misinterpretation of these results could lead to unnecessary risk reduction strategies, including unnecessary surgeries. Patients who are found to have a VUS are more likely to have residual cancer distress than patients who have a negative but uninformative result, but genetic counseling has been shown to help with their understanding of their and their family’s risk, helped them make medical decisions to assist in lowering their risk, and decreased worry (27).

## Results Disclosure and Post-Test Counseling

Post-test counseling should include an explanation of results, options for managing risk, as well as discussion of the implications for other family members. One of the distinguishing features of genetic medicine is that the discovery of a hereditary predisposition to cancer or other disease has implications not only for the individual tested, but also for their family members. The transmission of this information is generally accomplished by communication between the patient and each of his or her relatives. Although it is well recognized that family history impacts risk, there are barriers to patients disclosing their genetic testing results to their families. These include difficult or distant family relationships, lack of perceived usefulness, the serious nature of the message, and the concern that the message will be rejected (28, 29). In a study of 273 women assessed 4 months after BRCA1/2 result disclosure, demographic, health and test-related factors predicted communication of results to family members. Female relatives were more likely to be informed than males (23% of participants did not inform their father and 29% of brothers were not informed). Inconclusive results (negative without known familial mutation or variant of uncertain significance) were less likely to be conveyed than conclusive (positive or true negative) results. Women over the age of 40 were less likely to convey results to their parents than women under age 40. Age influenced communication to children, but most children were told (30). In another study, relatives of BRCA1/2 positive individuals were offered cost-free and confidential genetic counseling and testing. They were then surveyed about disclosure of their genetic testing results. Seventy-seven percent of those surveyed disclosed results to all at risk relatives. The disclosure of results to first degree relatives was higher than to second or third degree relatives. Disclosure rates to males and females were similar, but women who received the results were more likely to have genetic testing, as were individuals from the maternal lineage. Men were more likely to express difficulty discussing results and women were more likely to experience emotional distress associated with the results disclosure (31).

How can we as providers guide patients and facilitate intrafamilial communication? McClellan and colleagues conducted an exploratory study to assess resources to support patients and health-care providers, specifically addressing intrafamilial communication of cancer risk (32). Resources are provided by health-care professionals, health service organizations, and patient groups, but not all of the materials are always cohesive. Among health-care professionals, most of the resources acknowledge the importance to family members and may provide information regarding consequences to families, but do not often address the logistics of message delivery or provide tools or direction to aid in message delivery. These resources addressing logistic of message delivery and the provision of tools, such as sample letters, are more available through service organizations of patient groups. Overall, though, few resources are offered in support of intrafamilial communication, and further consideration of these complex issues is necessary to aid patients in sharing this complex information with their family members.

A randomized clinical trial conducted in the Netherlands assessed the use of the Informing Relatives Inventory, a battery of instruments measuring knowledge, motivation, and self-efficacy regarding disclosure of hereditary information to at-risk relatives, and found that it improved insight into patients’ barriers regarding result disclosure (33). Further research is needed to assess these barriers more completely, however, and provide insight into ways to assure that results disclosure occurs to all family members who might benefit from the information.

Ensuring that family members are made aware of the risk is not always easy, as patients are not legally required to disclose medical information to their relatives. In addition, the Health Insurance Portability and Accountability Act (HIPAA) prohibits health-care professionals from disclosing a patient’s information to third parties, including family members. When communication between patient and relatives fails, what is the responsibility of the provider? In some instances, the law mandates release of medical information to third parties (transmissible diseases, child abuse, domestic violence, or conditions that constitute a danger to public safety), but to date, genetic testing results have not been considered exempt from privacy laws. Even if not subject to HIPAA laws, disclosing genetic test results to all family members of an affected individual places an unrealistic burden on the practitioner. So while it is acknowledged that health-care providers have a duty to inform patients about the implications of test results for the family and should encourage the dissemination of this information, it is currently beyond the scope of practice for most providers. Certain circumstances may provide additional challenges. For example, what if the individual tested is deceased? What about children relinquished to adoption? Further discussion is necessary to determine the best way to assure that those who benefit from learning the family’s results can obtain the information necessary to address their own surveillance needs.

## Future Directions

There has been tremendous growth in the ability to test for potential hereditary causes of cancer, but the testing to date has largely been guided by family history. As we continue to personalize cancer care, BRCA testing or testing for other genes, will likely be performed for all patients newly diagnosed with breast cancer. There are already some therapeutic implications. Poly ADP-ribose polymerases (PARPs) are important components of the base excision repair pathway for single strand DNA breaks. PARP inhibitors, therefore, block this mechanism of DNA repair. These agents have shown the most promise in patients with breast or ovarian cancer who have germ line BRCA mutation or non-mutational functional defects in BRCA proteins (34, 35). Along similar lines, early data suggest that BRCA1 mutations result in increased sensitivity to DNA damaging chemotherapy, including platinum agents, and relative resistance to microtubule agents, such as taxanes (36–38). If additional data support the importance of BRCA mutation status to determine systemic therapy, this testing may be routinely performed at diagnosis. In the future, it seems likely that at least some genetic testing will be performed in all patients newly diagnosed with breast cancer.

Some argue that waiting until the family meets criteria for genetic testing is too late to identify a mutation in the family. The ideal time to identify risk is prior to a cancer diagnosis. A study published by Manchanda and colleagues evaluated cost-effectiveness of populations screening for BRCA mutations in Ashkenzi Jewish women compared with family history-based testing and found that this was highly cost-effective for women of Ashkenazi Jewish ancestry 30 years and older (39). Of course, testing in this specific population consists of testing for the three common mutations that cause 95% of cases of Hereditary Breast and Ovarian Cancer in Ashkenazi Jewish individuals. Also, the carrier frequency of mutations is 1/40 as opposed to 1/400–800 in non-Jewish individuals. At this time, population screening of non-Jewish individuals is not really feasible or cost-effective, but as the cost of testing continues to decrease, this will likely occur.

Finally, as we learn more about the implications of more moderate risk genes, the use of widespread panels will likely also increase and will likely also expand to routine testing of women. This will allow a better identification of those at risk and will likely allow better predictions of cancer risks and allow for earlier consideration of screening and preventive options.

In summary, genetic testing for breast cancer susceptibility has advanced tremendously, but we are only scratching the surface of our understanding of the role heredity plays in carcinogenesis. There is no doubt, however, that our knowledge will continue to grow allowing us to more accurately provide assessment of risk to all individuals, regardless of their family history.

## Author Contributions

The authors jointly conceptualized the article and discussed the aspects of the topic to be addressed. DA performed the primary literature review and first authored the paper. RP provided critical review.

## Conflict of Interest Statement

The authors declare that the research was conducted in the absence of any commercial or financial relationships that could be construed as a potential conflict of interest.
